# Complete mitochondrial genome of the Southern two-lined salamanders (*Eurycea cirrigera*)

**DOI:** 10.1080/23802359.2017.1413434

**Published:** 2017-12-08

**Authors:** XingRan Wang, Junkui Li, LiNa Zhang, HongQing Li, Kai Ma, Ying Han

**Affiliations:** a College of Animal Science and Technology, Northeast Agricultural University, Harbin, P.R. China;; b Department of Gynecology and Obstetrics, No.254 Hospital of PLA, TianJin, China

**Keywords:** Eurycea cirrigera, mitochondrial genome, gene

## Abstract

The complete mitochondrial genome of the southern two-lined salamanders (*Eurycea cirrigera)* was determined in this study. The complete mitogenome sequence of *E. cirrigera* is 16,759bp in length and contains 37 genes (13 protein coding genes (PGCs), two ribosomal RNA, 22 transfer RNA genes and one control region). This is the first time of the mitochondrial genome sequencing for *E. cirrigera.*

The southern two-lined salamander*, Eurycea cirngera* (Family Plethodontidae: Subfamily Spelerpinae), is a forest-dependent species with a wide distribution throughout the eastern United States extending west to the Mississippi River in Mississippi, Louisiana, Tennessee and the eastern edge of Illinois. Because it is such a wide-ranging species, geographic variation in life history traits is common (Petranka 1998). Genetic information would be useful for wild life conservation and forensics. However, researches of complete mitochondrial genome of *E. cirrigera* have not been reported. Therefore, we assembled and characterized the complete mitochondrial genome sequence (mitogenome) of *Eurycea cirrigera* in this study.

In this study, one adult was collected from Mississippi River in Louisiana(29°57′26.9″N 90°08′34.2″W) and used for the complete mitochondrial DNA sequencing. The specimen is stored in Northeast Agricultural University and its accession number is NEAUDK14628. Genomic DNA was extracted from muscle tissue using the standard proteinase K, phenol: chloroform: iso-amyl procedure (Liang et al. 1994). The mitogenome of *Eurycea bislineata* (AY728217.1) was employed as reference sequence. In order to amplify the target sequence, 26 primers were designed. With the aim of conserving the genetic resource of *E. cirrigera*, the assembly of sequencing result was performed using ContigExpess 9.0 software (New York, NY). Manual alignment of DNA sequence fragments were performed using DNASTAR 5.0 and compared with other Salamandridae species to determine the location of protein-coding genes. Using the program tRNAscan-SE 1.21 (http://lowelab.ucsc.edu/tRNAscan-SE) to identify the transfer RNA.

The complete mitogenome sequence of *E. cirrigera* is 16,759bp in length (GenBank accession number KY752074). It contains 13 protein-coding genes, two ribosomal RNA, 22 transfer RNA genes and one control region. The overall base composition is 33.02% for A, 22.75% for C, 13.65% for G, and 30.58% for T. Most of the genes are similar in length to their counterpart genes in other Salamandridae (Zhang et al. 2008; Chen et al. 2016). Meanwhile, most of the tRNA genes are coded expect for tRNA^Pro^, tRNA^Gln^, tRNA^Ala^, tRNA^Asn^, tRNA^Cys^, tRNA^Tyr^, tRNA^Ser^, tRNA^Thr^ and tRNA^Glu^. Most of these PCGs initiate with an ATG as start codon except for COX1 which begins with GTG. The length of the 12SrRNA and 16SrRNA genes in the *E. cirrigera* mitochondrial genome are 903bp and 1494bp, respectively. The gene composition and arrangement are identical to those of most amphibians (Jiang et al. 2016; Liu et al. 2017).

To validate the phylogenetic position of *E. cirrigera*, the genomewide alignment of 27 mitochondrial genomes in Salamandridae was construction by MEGA 6.06 resulting in 16,759 characters. Phylogenetic relationships obtained with the maximum-likelihood approach were identical to those of the Bayesian analysis (Posada and Buckley 2004).Bayesina phylogenetic analysis was conducted with MrBayes 3.2.5 (Ronquist and Huelsenbeck 2003). As shown in the phylogenetic tree (Figure 1), *E. cirrigera* sequence was clustered in genu Plethodontidae, including *Eurycea bislineata, Plethodon cinereus, Plethodon elongates, Plethodon Petraeus* and *Ensatina eschscholtzii. Plethodontidae* were distinctly separated from other families in Salamandridae.

**Figure 1.**
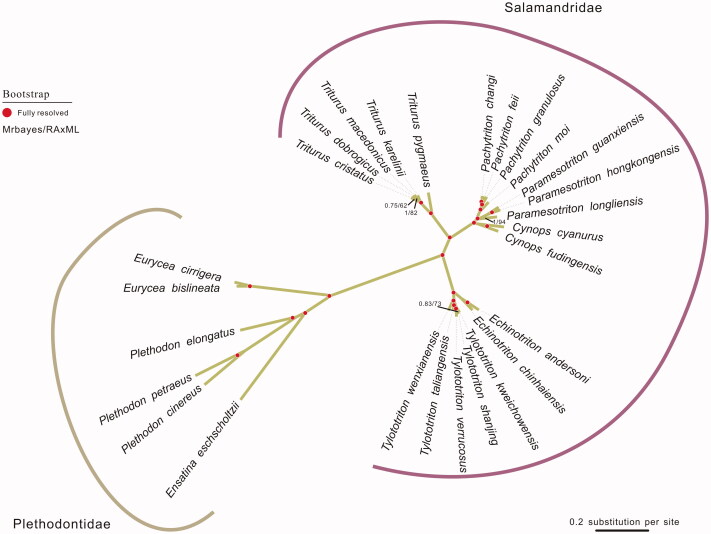
The phylogenetic tree of the 27 species from Salamandridae was constructed based on complete mitochondrial genome data. The analysed species and corresponding GenBank accession numbers are as follows: *Cynops cyanurus* (NC_032314.1), Cynops fudingensis (NC_032315.1), *Echinotriton andersoni* (NC_017870.1), *Echinotriton chinhaiensis* (NC_032068.1), *Ensatina eschscholtzii* (NC_006328.1), *Eurycea bislineata* (AY728217.1), *Eurycea cirrigera* (KY752074), P*achytriton changi* (NC_032313.1), *Pachytriton feii* (KT899979.1), *Pachytriton granulosus* (KX021918.1), *Pachytriton moi* (NC_032312.1), *Paramesotriton guanxiensis* (KX021916.1), *Paramesotriton hongkongensis* (AY458597.1), *Paramesotriton longliensis* (NC_032310.1), *Plethodon cinereus* (AY728232.1), *Plethodon elongates* (AY728223.1), *Plethodon Petraeus* (AY728222.1), *Triturus cristatus* (NC_015790.1), *Triturus dobrogicus* (NC_015791.1), *Triturus karelinii* (NC_015792.1), *Triturus macedonicus* (NC_015794.1), *Triturus pygmaeus* (NC_015796.1), *Tylototriton kweichowensis* ( NC_029231.1), *Tylototriton shanjing* ( KR154461.1), *Tylototriton taliangensis* (KP979646.1), *Tylototriton verrucosus* (NC_017871.1), *Tylototriton wenxianensis* ( NC_027507.1).
